# EARL compliance measurements on the biograph vision Quadra PET/CT system with a long axial field of view

**DOI:** 10.1186/s40658-022-00455-1

**Published:** 2022-04-08

**Authors:** George A. Prenosil, Michael Hentschel, Thilo Weitzel, Hasan Sari, Kuangyu Shi, Ali Afshar-Oromieh, Axel Rominger

**Affiliations:** 1 Department of Nuclear Medicine, Inselspital Bern, Bern University Hospital, University of Bern, 3010 Bern, Switzerland; 2 Advanced Clinical Imaging Technology, Siemens Healthcare AG, Lausanne, Switzerland

**Keywords:** EARL, Exposure, Image reconstruction, Long axial field of view, Quantitative ^18^F PET/CT

## Abstract

**Background:**

Our aim was to determine sets of reconstruction parameters for the Biograph Vision Quadra (Siemens Healthineers) PET/CT system that result in quantitative images compliant with the European Association of Nuclear Medicine Research Ltd. (EARL) criteria. Using the Biograph Vision 600 (Siemens Healthineers) PET/CT technology but extending the axial field of view to 106 cm, gives the Vision Quadra currently an around fivefold higher sensitivity over the Vision 600 with otherwise comparable spatial resolution. Therefore, we also investigated how the number of incident positron decays—i.e., exposure—affects EARL compliance. This will allow estimating a minimal acquisition time or a minimal applied dose in clinical scans while retaining data comparability.

**Methods:**

We measured activity recovery curves on a NEMA IEC body phantom filled with an aqueous ^18^F solution and a sphere to background ratio of 10–1 according to the latest EARL guidelines. Reconstructing 3570 image sets with varying OSEM PSF iterations, post-reconstruction Gaussian filter full width at half maximum (FWHM), and varying exposure from 59 kDecays/ml (= 3 s frame duration) to 59.2 MDecays/ml (= 1 h), allowed us to determine sets of parameters to achieve compliance with the current EARL 1 and EARL 2 standards. Recovery coefficients (RCs) were calculated for the metrics RC_max_, RC_mean_, and RC_peak_, and the respective recovery curves were analyzed for monotonicity. The background’s coefficient of variation (COV) was also calculated.

**Results:**

Using 6 iterations, 5 subsets and 7.8 mm Gauss filtering resulted in optimal EARL1 compliance and recovery curve monotonicity in all analyzed frames, except in the 3 s frames. Most robust EARL2 compliance and monotonicity were achieved with 2 iterations, 5 subsets, and 3.6 mm Gauss FWHM in frames with durations between 30 s and 10 min. RC_peak_ only impeded EARL2 compliance in the 10 s and 3 s frames.

**Conclusions:**

While EARL1 compliance was robust over most exposure ranges, EARL2 compliance required exposures between 1.2 MDecays/ml to 11.5 MDecays/ml. The Biograph Vision Quadra’s high sensitivity makes frames as short as 10 s feasible for comparable quantitative images. Lowering EARL2 RC_max_ limits closer to unity would possibly even permit shorter frames.

## Background

Clinical positron emission tomography (PET) systems have found a widespread use in many fields of diagnostics and follow-up care. This success is at least partially owed to the technological progress PET has experienced since its debut roughly 45 years ago [1]. In combination with computed tomography (CT), PET/CT became an ever-evolving instrument for quantitatively measuring the spatial distribution of positron emitting tracers [2]. Continuous improvement in detector technology, such as employing small and fast lutetium oxyorthosilicate (LSO) crystals for photon conversion and fast electronics, increased spatial resolution of molecular imaging and allows time-of-flight (TOF) measurements. The TOF technology increased signal-to-noise ratio (SNR) [3–5] and thus the apparent sensitivity. Recent development in detector technology includes the replacement of analog photomultiplier tubes with digital silicon photomultipliers, giving rise to so-called digital PET/CT systems. Their higher gain, better coverage of the LSO crystals, better energy resolution and faster read-out made digital PET/CT systems generally outperform their analog forerunners in lesion detection and acquisition duration [6–8]. On the software side, modern nonlinear reconstruction algorithms use resolution recovery techniques for reducing the partial volume effect (PVE) [9, 10]. The PVE, inherent to all imaging devices, blurs the image and impedes tracer uptake quantification in small objects.

Current state of the art is digital total-body PET/CT systems with a long axial field of view (AFOV) such as the Biograph Vision Quadra (Siemens Healthineers) and the uEXPLORER (United Imaging) [11]. The Biograph Vision Quadra essentially is comprised of an axial concatenation of the equivalent of four Biograph Vision 600 scanners [12], providing an AFOV of 106 cm. Besides enabling the simultaneous imaging of distant anatomical regions, the long AFOV increases count rate and thus sensitivity. The Quadra’s current clinical high sensitivity mode, where not all lines of response are used to form the PET image, increases sensitivity by a factor of five when compared to the preceding Biograph Vision 600 [13, 14]. A future upgrade to an ultra-high sensitivity mode (e.g., MRD 322, *c.f.* below), with all lines of response used, will raise sensitivity even further. This sensitivity increase allows either better images, a reduction in patient radiation dose, or shortening the acquisition duration [15]. The latter aspect makes the Biograph Vision Quadra an ideal imaging tool in dynamic total-body studies with high spatial resolution.

However, differences in instrumentation and image reconstruction between the commercially available PET/CT systems impact image comparability. Hence, data acquired on differing PET/CT systems add statistical spread to multicenter clinical studies [16, 17], which in its turn renders quantitative tracer uptake measurements a poorer diagnostic indicator [18]. Therefore, efforts for establishing PET/CT comparability have been undertaken. Since 1994, National Electrical Manufacturers Association (NEMA) performance measurements evaluate comparatively PET/CT systems with standardized procedures [19], providing data on a PET system’s imaging characteristics. The NEMA NU 2 standards publications are updated regularly, mirroring the technological progress in the field [20]. Even though the NEMA standards provide various metrics for quantifying and comparing PET/CT performance, differences in image formation remained to be addressed. Therefore, the method of transconvolution has been introduced that recasts images acquired on different PET/CT systems as if they had all been acquired on the same PET system [21] or with the same positron emitter [22, 23]. Another approach uses adapted post-reconstruction filtering for data harmonization [24], with the drawback of downgrading the better PET/CT systems within a consortium. Finally, the European Association of Nuclear Medicine Research Ltd. (EARL) initiative introduced complete harmonizing strategies [25, 26] in their accreditation program. EARL accreditation is meant especially for participating in multicenter clinical studies [27], where it guarantees comparable data sets even between different generations of PET/CT systems or different manufacturers [28].

Obtaining an EARL accreditation requires the applying PET site to perform two phantom measurements while following a prescribed image acquisition protocol: the first acquisition uses a homogenous cylinder phantom filled with a known activity concentration to verify the correct cross-calibration of the PET/CT system [29] with the hot-lab’s dose calibrator. The second acquisitions use an IEC body phantom for assessing image quality with quantitative image metrics. These metrics quantify the measured activity concentration within the phantom’s spheres and are otherwise in clinical use for lesion characterization. All image metrics are then expressed in relation to the filled activity concentration as recovery values, which must be kept within specified limits through the accordant choice of image reconstruction parameters.

Reflecting advances in PET instrumentation such as increased spatial resolution, TOF, and point spread function (PSF) image reconstructions that result in higher signal recoveries, EARL has complemented its original EARL1 standard with a second EARL2 standard [30]. Compared to EARL1, EARL2 demands higher recovery values for smaller spheres and generally has a narrower acceptance band [31, 32]. However, EALR2 has never been derived nor assessed from measurements on total-body PET/CT systems. Both EARL1 and EARL2 standards are currently in effect for fluorine-18 measurements, and PET/CT sites are free to follow either one.

Although PET performance measurements result in various metrics for comparing imaging properties and sensitivity between different PET/CT systems [20], optimal clinical acquisition durations are not specifically addressed in NEMA image quality measurements nor in the EARL guidelines [25, 26]. EARL guidelines demand clinically relevant acquisition durations of five minutes per bed position, but make no considerations to a PET/CT system’s sensitivity. Work by Kaalep et al. [31] describes the effect of long and short acquisitions on PET data harmonization using the EARL standards, but no official EARL recommendation for image exposure resulted thereof. As shown for the Biograph Vision 600 [8], knowledge of the required minimal exposure on a given PET/CT system is important for estimating the shortest feasible acquisition duration or the minimum injected dose, while simultaneously retaining reproducible and comparable PET/CT images. Pilz et al*.* [33] demonstrated a certain tolerance of EARL compliant FDG PET/CT measurements to image noise, reducing clinical acquisition duration down to 57 s per bed position on their 3D TOF Ingenuity TF PET/CT system (Philips, Cleveland, OH). With image reconstruction parameters optimized for low exposures, even shorter EARL compliant acquisition durations can be possible.

This work aims to determine sets of reconstruction parameters for the Biograph Vision Quadra PET/CT system that result in EARL compliant images. We have previously defined exposure in PET/CT as the time-activity-product, e.g., the number of incident counts the systems sees, to demonstrate its impact on metrics for textural features in PET images [34]. This metric has been useful for comparing different PET/CT systems [35]. Here, we also explore how exposure affects EARL image quality compliance on a PET/CT system with a long AFOV. This question is especially relevant for dynamic studies, where tracer uptake changes during acquisition, and potentially degrades comparability of measurement points within a time activity curve.

From activity recovery measurements, we deduced image reconstruction parameter sets that resulted in EARL1 and EARL2 compliance for ^18^F [26, 27] under varying exposure for the Biograph Vision Quadra. This allowed us to formulate minimal and maximal exposure regimes for this new PET/CT system. Furthermore, monotonicity of recovery curves was analyzed.

## Material and methods

The aim of this study was to identify a complete image parameter space for obtaining quantitative, EARL compliant PET/CT images for the Biograph Vision Quadra. For this purpose, the examined parameters were the number of reconstruction iterations, post-reconstruction Gaussian filter full width at half maximum (FWHM), and exposure. All measurements were performed on a PET phantom commonly used for assessing image quality.

### Phantom measurements

A NEMA IEC body phantom [20, 36] with six hollow spheres of 10, 13, 17, 22, 28, and 37 mm internal diameter was filled with an aqueous solution of [^18^F]FDG to a sphere to background activity concentration ratio of 10 to 1, using a scale and a dose calibrator (ISOMED 2010, NUVIA Instruments GmbH, Germany). At the start of PET acquisition, the background activity concentration was 1.97 kBq/ml; the respective foreground activity concentration was 19.7 kBq/ml. The six spheres were positioned at a radius of 57.2 mm around the phantom center, surrounding a cold lung insert of 50 mm diameter filled with polystyrene beads. The phantom was placed with the spheres in the center of the FOV and axially aligned with the scanner as required by the NEMA NU 2-2018 protocol [20].

List mode data were acquired on the Biograph Vision Quadra lasting for one hour for one bed position. Using these data, images were reconstructed using a software prototype for image reconstruction (Siemens Healthineers) using the PSF + TOF (TrueX) algorithm. Multiple images were reconstructed using frame durations of 3 s, 10 s, 30 s, 60 s, 180 s, 300 s, 600 s, and 3600 s. The final one-hour long image reconstruction served as a best, low noise acquisition while the five minutes frame duration was the current EARL specification recommended for clinical images [26]. The different acquisition durations resulted in exposures of 0.06, 0.2, 0.6, 1.2, 3.5, 5.8, 11.5, and 59.2, all values given in units of MDecays/ml. All images were reconstructed into a 440 × 440 matrix, with 1.65 mm slice thickness for an isometric voxel spacing. In 510 reconstructions per frame duration, we then systematically varied the Gaussian post-reconstruction filter FWHM from 0 mm (all pass) to 10 mm in increments of 0.2 mm and the number of reconstruction iterations from one to ten in increments of one to identify the particular parameter set that resulted in EARL1 and EARL2 compliance for a given exposure. The number of iterative subsets was kept constant at five, because reconstructions with the same iteration-subsets products result in similar images [37]. All image reconstructions were performed offline on a HP Z8 G4 workstation (HP Inc., Palo Alto, CA, USA), equipped with a 3.1 GHz Intel® Xeon® Gold 6254 CPU, and running the scriptable software prototype dedicated to image reconstruction.

The PET data were corrected for randoms, attenuation, scatter, and decay. The CT scans for attenuation correction were acquired with 120 keV tube voltage, 80 mAs tube current and with 0.8 pitch. The CT images were reconstructed into a 512 × 512 matrix, with a 5 mm collimation, and with an axial increment of 1.65 mm for isotropic PET voxels.

### Definitions

#### Exposure

Exposure *E* was defined as the total number of expected decay events per volume during acquisition time *Δt*, and starting with a mean initial activity concentration *AC*_*0*_. When using a short-lived isotope, such as ^18^F, exposure must be calculated as the integral over all decays encountered, instead as just the product of activity concentration and acquisition duration [34]:

1 $$E=\underset{0}{\overset{\Delta t}{\int }}{AC}_{0}*{2}^{-\frac{t}{{t}_{1/2}}} dt$$

The integral resolves into

2 $$E=\frac{{{t}_{1/2}*AC}_{0}}{\mathrm{ln}2}*\left(1-{2}^{-\frac{\Delta t}{{t}_{1/2}}}\right)$$

Here, t_1/2_ is 109.77 min, the half-life of ^18^F. Exposure was always defined for the foreground activity concentration, i.e., *AC*_*0*_ in the phantom spheres. In clinical images, exposure would thus normally be calculated for the investigated lesions and not for the background signal. It must be noted that our simple exposure metric does not take into account a count rate dependency of image noise. However, for our purpose of comparing different frame durations at clinical activity concentrations, the noise-equivalent count rate (NECR) curve of the Biograph Quadra is sufficiently linear [14]. Furthermore, the NECR concept does not capture a PET/CT system’s image noise non-stationarity, nonlinearity and spatial frequency dependency.

#### Quantitative image metrics

All measurements obtained from the spheres were normalized to the 19.74 kBq/ml ^18^F filled in at the start of the PET acquisition to obtain recovery coefficients (RCs). In this work, phantom PET/CT images were analyzed for RC_max_, RC_peak_, and RC_mean_ according to the current EARL guidelines [24, 25]. The RC_max_ metric was the highest value found within the phantom sphere location divided by the actual activity in the sphere. The RC_mean_ metric was calculated from the average value in an automatically grown volume of interest (VOI) around the RC_max_ location, which only included voxels with a value equal or greater than 50% of the maximal value found in said VOI (VOI A_50_) [38, 39].

To achieve EARL compliance, recovery coefficients for RC_max_, RC_peak_, and RC_mean_ had to be within the limits for all phantom spheres published in the EARL guidelines for ^18^F PET/CT on July 2020 [30]. Table 1 shows the currently valid EARL1 and EALR2 limits.

**Table 1:** EARL1 and EARL2 RC limits. *At the time of this work, RC_max_ for total-body scanners was under investigation. ** RC_peak_ limits were under revision. Table adapted from the EARL webpage [30]

NEMA IEC phantom spheres			^18^F standard 1 RC limits		^18^F standard 2 RC limits		
Sphere *i*	Diameter (mm)	Volume (mL)	RC_max_	RC_mean_	RC_max_*	RC_mean_	RC_peak_**
1	37	26.52	0.95–1.16	0.76–0.89	1.05–1.29	0.85–1.00	0.90–1.10
2	28	11.49	0.91–1.13	0.72–0.85	1.01–1.26	0.82–0.97	0.90–1.10
3	22	5.57	0.83–1.09	0.63–0.78	1.01–1.32	0.80–0.99	0.90–1.10
4	17	2.57	0.73–1.01	0.57–0.73	1.00–1.38	0.76–0.97	0.75–0.99
5	13	1.15	0.59–0.85	0.44–0.60	0.85–1.22	0.63–0.86	0.45–0.70
6	10	0.52	0.34–0.57	0.27–0.43	0.52–0.88	0.39–0.61	0.27–0.41

The metrics RC_max_, RC_peak_ and RC_mean_ from phantom data for every sphere *i* were then plotted against sphere diameter to obtain recovery curves. Furthermore, the relative coefficient of variation (COV) of the image signal was calculated from nine planar background regions of interest (ROIs) of 900 mm^2^ each and according to reference [40]. The COV had to be below 0.15 for an EARL compliant image noise.

### Data analysis

#### EARL compliance analysis of recovery curves

We introduced the normalized root-mean-square error (nRMSE) to assess quantitatively EARL compliance of recovery curves. The nRMSE incorporated the distance *d*_*i*_ of a recovery curve value *RC*_*i*_ to the average EARL compliance value *RC*_*average*_ as residuals for every sphere *i*. *RC*_*average*_ value arose from the average of the low and high EARL limits for a given recovery metric at position *i* in the RC. To arrive at a comparable measure of compliance, regardless of the recovery metric used, *d*_*i*_ was normalized to the relative width of the EARL limits band for sphere *i*.

3 $${d}_{i}=\frac{{RC}_{i}-{RC}_{average}}{\left({EARL}_{high,i}-{EARL}_{low,i}\right)}, \,and\, {RC}_{average}=\frac{\left({EARL}_{high,i}-{EARL}_{low,i}\right)}{2}$$

From *d*_*i*_, nRMSE was calculated:

4 $$nRMSE=\sqrt{\frac{1}{6}\sum {d}_{i}^{2}}$$

For a concise compliancy report, the nRMSE values for the recovery metrics RC_max_ and RC_mean_ were aggregated into a single value according to the formula below:

5 $$nRMSE=\sqrt{{(nRMSE}_{RCmax}^{2}+{nRMSE}_{RCmean}^{2})/2}$$

Because at the time of this work, RC_peak_ was under revision for EARL2, we analyzed this metric separately for EARL2 compliancy, without aggregating it with the other two recovery values. This allowed determining the exact combination of optimal image reconstruction parameters for EARL2 compliant RC_peak_ values, and it should help evaluating this parameter in future.

#### Statistical data analysis

The Spearman’s rank correlation coefficient *ρ* was used as a metric for monotonicity of recovery curves (RC vs. sphere diameter), with

6 $$\rho \in {\mathbb{R}}|-1\le \rho \le 1$$

A *ρ* = -1 or *ρ* = 1 indicates perfect decreasing or increasing monotonicity, whereas *ρ* = 0 means complete absence of monotonicity. Recovery curve monotonicity was not an EARL criterion but was added here as a quality metric for recovery curves.

#### Software

The phantom data were analyzed using an in-house software which itself was realized on our rapid application development framework written in Java and Prolog. This software used scriptable batch processing for the automated analysis of 3570 image reconstructions obtained by varying iterations, Gauss FWHM and exposure. The software ran on a HP Z640 workstation (HP Inc., Palo Alto, CA, USA).

## Results and discussion

### EARL compliance testing

**Fig. 1:**
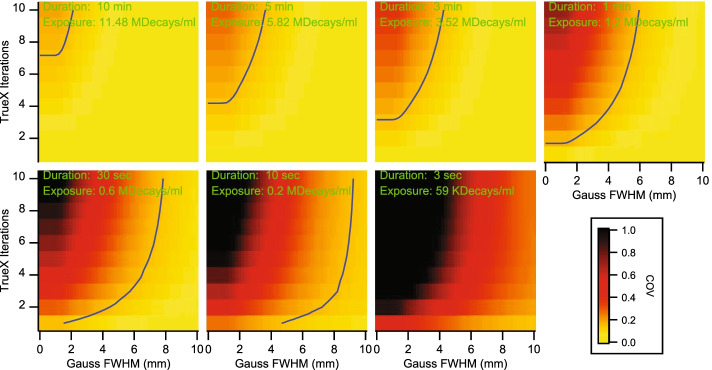
Image background COV in reconstruction parameter space and for different exposures with EARL1 COV cut-off values at 0.15 (blue contour)

Figure 1 shows the COV obtained from the image background with varying reconstruction parameters. Except for the 3 s acquisitions, all frame durations had parameters combinations that resulted in EARL compliant image noise with COV values below 0.15. The one-hour long frames had COV values below 0.15 for all investigated reconstruction parameter combinations.

**Fig. 2:**
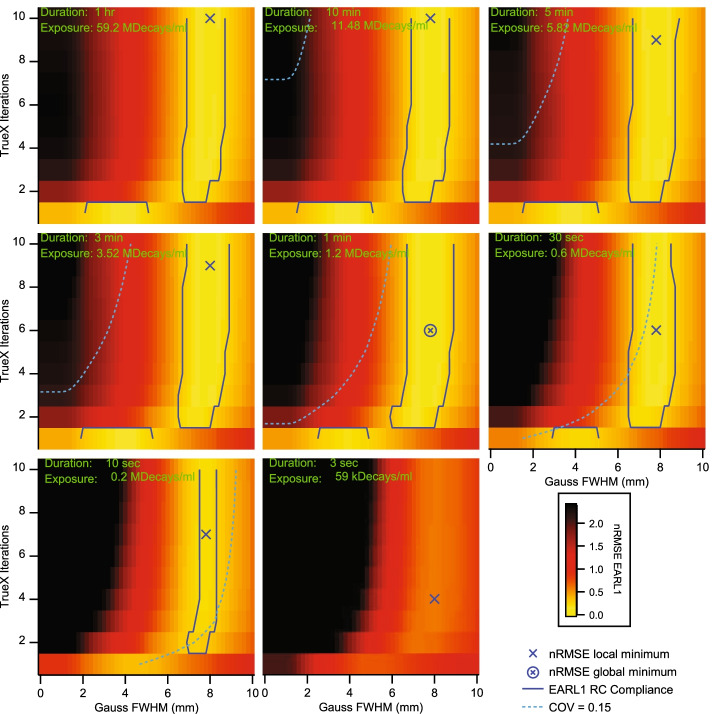
Normalized RMSE for RC_max_ and RC_mean_ in reconstruction parameter space. Data sets within the blue contour passed the current EARL1 recovery curve limits; each panel represents a different exposure. The light blue dotted lines mark the EARL COV cut-off values from Fig. 1; compliant data sets were found on the dotted line’s right side. The blue cross marks the local nRMSE minima; the cross in the circle marks the global nRMSE minimum. Panels are shown in order of decreasing exposure

Plotting in Fig. 2 the total nRMSE as a function of the reconstruction parameter space for reconstructions that passed EARL1 compliance revealed the optimal reconstruction parameter combinations for EARL1 compliant PET/CT images at different exposures.

The Biograph Vision Quadra achieved exposure stable EARL1 compliance within a Gauss FWHM corridor ranging from 6.8 to 8.6 mm and for two to ten iterations in most frames (Blue contours in Fig. 2). In the 10 s frame, the accepted Gauss FWHM corridor narrowed down to between 7.6 and 8.2 mm. The lowest nRMSE relative to the EARL1 limits in the 10 s images had a value of 0.22 and was found at 7 iterations, 5 subsets and 7.8 mm Gauss filtering. However, this reconstruction had a background COV over 0.15, and it would therefore not pass EARL compliance due to excessive image noise. The overall lowest nRMSE value of 0.075 and a COV of 0.08 was found in the 1 min frame for 6 iterations, 5 subsets and 7.8 mm Gaussian FWHM, suggesting these being the most optimal and stable image reconstruction parameters for EARL1 compliance with acquisition durations of 30 s or more. The 10 s frames had only a very narrow acceptance band at 2 iterations, 5 subsets, and 7.6 mm Gauss filtering when regarding recovery curve and noise (COV) limits.

**Fig. 3:**
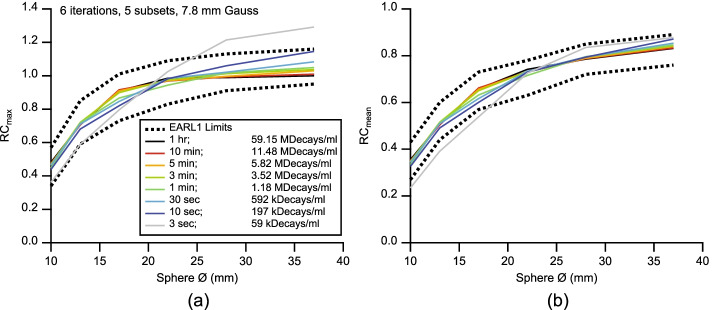
Optimal EARL1 compliant RC_max_ (**a**) and RC_mean_ (**b**) recovery curves for different exposures resulting from 6 iterations, 5 subsets, and 7.8 mm Gauss FWHM. Legend applies to both panels

Figure 3 shows the recovery curves resulting from the optimal image reconstruction parameters for all analyzed frames in relation to the respective EARL1 limits. It was usually the largest sphere that would express the highest image noise under low exposure regimes, with RC_max_ values above the specified limits (Fig. 3a).

**Fig. 4:**
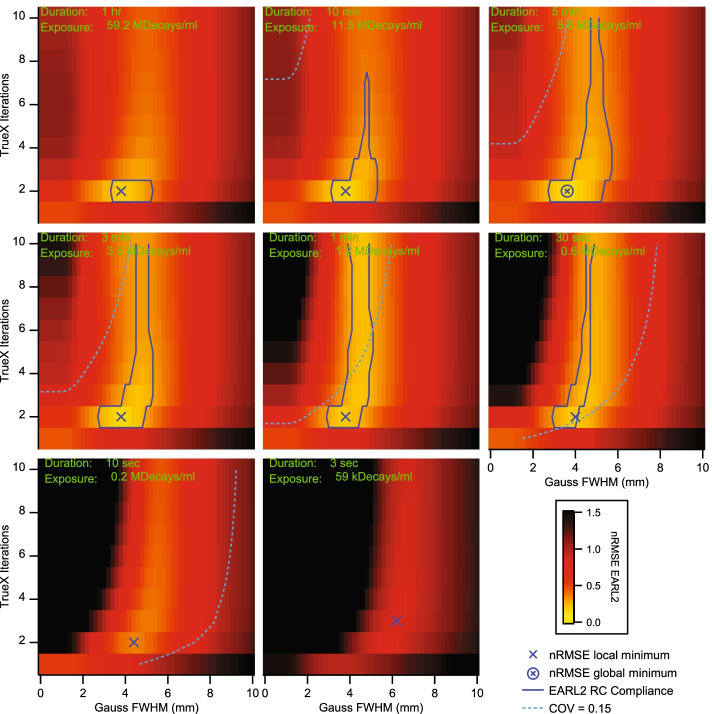
Normalized RMSE for RCmax and RCmean in reconstruction parameter space. Data sets within the blue contour passed the current EARL2 recovery curve limits; each panel represents a different exposure. The light blue dotted lines mark the EARL COV cut-off values from Fig. 1; compliant data sets were found on the dotted line’s right side. The blue cross marks the local nRMSE minima; the cross in the circle marks the global nRMSE minimum. Panels are shown in order of decreasing exposure

When analyzing the same image reconstructions for EARL2 compliance, it became clear that RCs from frames below 30 s and above 10 min were outside the specified EARL limits from Table 1. This translated to a minimal exposure of 0.6 MDecays/ml and to a maximal exposure of 11.5 MDecays/ml for EARL2 compliance without considering any noise limits. Figure 4 shows the total nRMSE from Eq. 5 for RC_max_ and RC_mean_ as a function of the number of iterations and Gauss FWHM concerning the EARL2 standards. The most stable image reconstructions regarding exposure were achieved with 2 iterations, 5 subsets, and a 3.6 mm Gauss FWHM. This reconstruction parameter combination resulted in a minimal nRMSE of 0.14 and a COV of 0.08 in the 5 min frame. In frames with longer exposures, recovery curves showed an increasing non-monotonicity, and the 3 sec frames always had COV values above 0.15 for reconstructions that were otherwise within the EARL2 recovery curve limits.

**Fig. 5:**
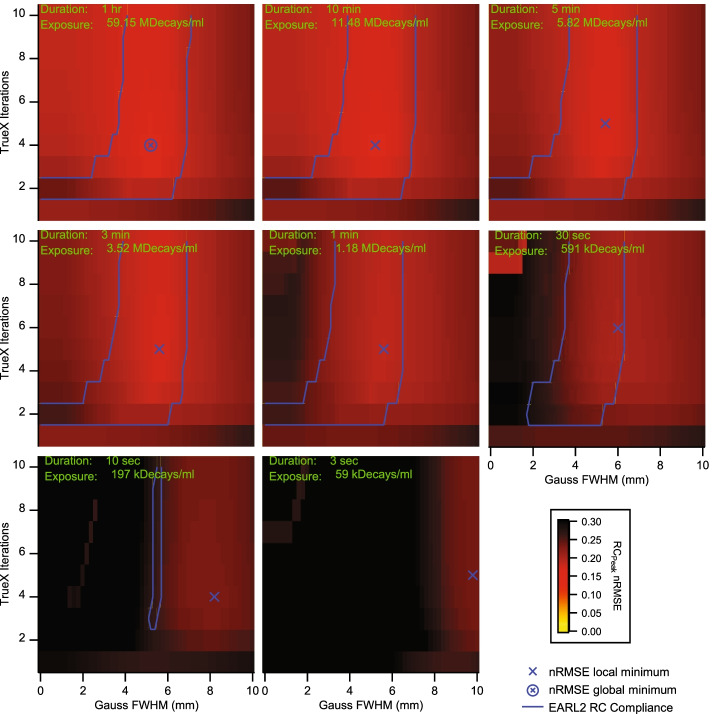
Normalized RMSE for RC_peak_ in reconstruction parameter space. Data within the blue contours passed the current EARL2 limits. The blue cross marks the local nRMSE minima; the cross in the circle marks the global nRMSE minimum. Panels are shown in order of decreasing exposure. The upper left corner of the 30 s panel was excluded from the nRMSE minimum consideration for reasons of relevance

When additionally analyzing RC_peak_ for EARL 2 compliance, it could be shown that this metric was always within EARL2 limits for a broad range of reconstruction parameters (Fig. 5). In the 10 s frames, compliance was restricted to a Gaussian FWHM of around 5.6 mm, while the 3 s frames were never EARL2 compliant. Similar to the other metrics, RC_peak_ was never within EARL2 limits for images reconstructed with only one iteration, and a minimum of three iterations was needed in the 10 s image. Minimal nRMSE values for RCpeak were found in most frames at 4 iterations, 5 subsets, and around a Gauss FWHM of 5.2 mm, and the minimal value in the frames decreased with increasing exposure. We conclude that for two or more iterations the compliance relevant metrics were RC_max_ and RC_mean_.

The nRMSE metric was not intended for general use with EARL recovery curves. It was solely conceived as a tool to quantify EARL compliance in a most meaningful way and to supplement but not to supplant the original digital EARL compliance statement. As long as all the relevant image metrics were within the stated EARL limits, nRMSE values for EARL compliant measurements could be higher compared to some measurements not passing EARL compliance. A good example for this situation is the 10 min frames panel in Fig. 4. However, low nRMSE values helped to point out exposure invariant recovery parameter combinations for quantitative PET/CT.

### Recovery curve monotonicity

**Fig. 6:**
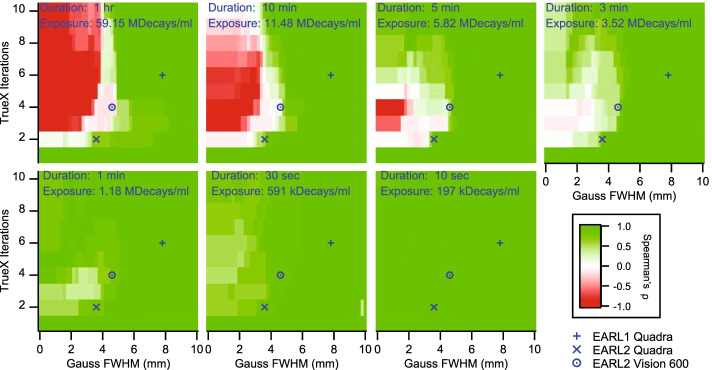
Monotonicity of RC_max_ recovery curves: Shown is the Spearman’s ρ as a function of number of TrueX iterations and post-reconstruction Gauss FWHM for different exposures. The markers designate the herein proposed reconstruction parameters for EARL1, EARL2, and EARL2 Vision compliant data. All 3 s frames displayed monotonically rising RC_max_ recovery curves

Recovery coefficients stay ideally as close as possible to unity, with the unavoidable drop for sphere sizes approaching the Nyquist limit, *i.e.,* the minimal object size that can be quantitatively sampled. Furthermore, while ideal recovery curves would be monotonic, real recovery curves recorded on modern PET/CT systems with nonlinear reconstruction algorithms are sometimes inflated for certain sphere sizes [41, 42]. Actually, the EARL2 limits intended for newer PET/CT systems even reflect this fact [32]. However, recovery curve non-monotonicity can potentially have clinical implications, e.g., when a therapy-induced shrinkage of a lesion might be actually interpreted as increased tracer uptake. Because RC_max_ was overall the most non-monotonous metric, we analyzed only RC_max_ recovery curves for monotonicity. The monotonicity value provided an additional criterion for selecting optimal sets of reconstruction parameters and exposures in Fig. 6.

While the above-suggested EARL1 compliant reconstruction parameter combinations always resulted in monotonic recovery curves, EARL2 compliant parameters yielded more often non-monotonic recovery curves (ρ ≈ 0). From our data in Figs. 3, 4, and 5, we therefore recommend a compromise of 2 iterations, 5 subsets and a post-reconstruction Gauss FWHM of 3.6 mm for monotonic EARL2 compliant recovery curves over most exposures. RC_max_ recovery curves from 1 min frames or shorter were generally monotonically rising (ρ > 0), as image noise was a function of sphere size. Higher exposures with higher iteration numbers and low post-reconstruction filtering tended to result in monotonically falling RC_max_ curves (ρ < 0). Here, the Biograph Vision Quadra over-iterated the smaller spheres, raising RC_max_ values above unity. The PSF reconstruction is essentially a deconvolution algorithm, where de-blurring of objects sized similarly to the system’s PSF can lead to overestimation of activity. This amplification effect increases with the number of iterations [43].

**Fig. 7:**
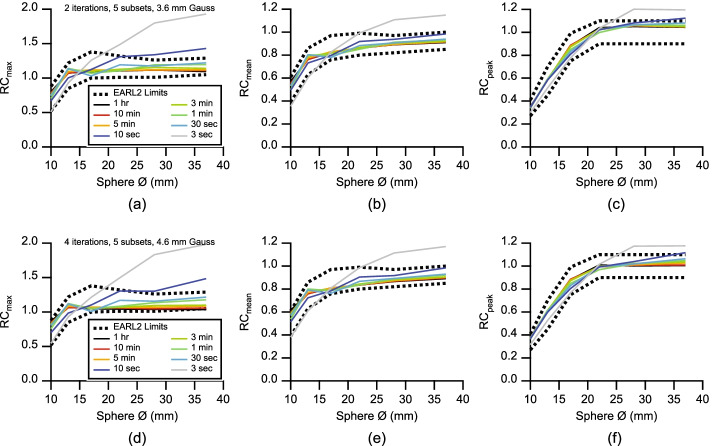
Upper row: Recovery curves calculated from RC_max_ (**a**) RC_mean_ (**b**) and RC_peak_ (**c**) for different exposures resulting from 2 iterations, 5 subsets, and 3.6 mm Gauss FWHM with respect to EARL2 limits. Lower row: recovery curves calculated from RC_max_ (d) RC_mean_ (**e**) and RC_peak_ (**f**) for different exposures resulting from 4 iterations, 5 subsets, and 4.6 mm Gauss FWHM with respect to EARL2 limits. Legend applies to all panels

Figure 7a–c shows the recovery curves resulting from the above-suggested image reconstruction parameters for all analyzed frames together with the respective EARL2 limits. Also shown are recovery curves reconstructed with parameters that are closer to the values suggested previously for the Biograph Vision 600 [8], a technologically related PET/CT system. These reconstruction parameters resulted in slightly less exposure robust recovery curves, only passing EARL2 compliance between 1 and 5 min exposure times (Fig. 7d–f).

#### Final discussion

It was apparent that lowering exposure increased RC_max_ in the largest spheres and decreased RC_max_ in the smallest spheres. The other two metrics, RC_mean_ and RC_peak_, were more robust in terms of exposure and monotonicity. When EARL2 compliance was not achieved, it was usually RC_max_ in the largest sphere not passing the EARL2 requirements. It is also noteworthy that in many high exposure reconstructions, RC_max_ was close to unity for the two largest spheres, indicating a low-noise image. Nevertheless, this resulted in non-compliance, as EARL2 does not allow an RC_max_ at unity for the three largest spheres (*c.f.* Table 1), instead mandating a certain presence of image noise. For example in the failed 4 iterations, 5 subsets, 4.6 mm Gauss FWHM 1 h acquisition in Fig. 7, RC_max_ was with 1.04 already below EARL2 limits for the largest sphere. A 1 h acquisition is certainly clinically impractical, but it gives us prospects on possible future gains in PET/CT sensitivity. Therefore, not having to introduce artificial image degradation to PET/CT systems with a long AFOV certainly warrants a currently ongoing re-evaluation of EARL2 limits.

The apparent paradoxical behavior of the large objects being more susceptible to image noise is vested in image count statistics: in imaging, the SNR measured within an imaged volume depends on the imaging system’s sensitivity *s* and on the number of available incident quanta from activity *A*, *i.e.,* exposure *E*. This gives the well-known SNR-exposure relationship:

7 $$SNR\propto \sqrt{s*A*\Delta t}\,\,\mathrm{ with }\,\,A*\Delta t=E$$

Even though the incident quanta strictly follow a random Poisson process, the intensity distribution that makes up the final PET image depends on the number of detected quanta and the chosen reconstruction method: while for filtered back projection, a Gaussian distribution can be assumed, images reconstructed with OSEM and PSF show lognormal [44, 45] or gamma-distributions [46]. Decreasing exposure broadens intensity distributions and gives rise to tail-heavy distributions in OSEM and PSF reconstructions [34]. RC_max_, being the supremum in the intensity probability distribution of the PET image, is therefore expected to rise with decreasing exposure. For the same reasons, object size correlates positively with RC_max_: the probability for finding higher RC_max_ becomes higher within larger sets of voxels. On the other hand, with object size falling below a critical value relative to the system’s PSF, the lower background noise from the spill-in of background intensity can dominate over object noise. In small objects, the PVE spills some of its noise out into the background, and a lower RC_max_ can therefore be expected in frames of shorter durations. It must be noted that in PET/CT systems with TOF, the effect of object size on RC_max_ might be somewhat diminished, as relative gains in apparent sensitivity from TOF are proportional to twice the object diameter [3]. The use of COV cut-off values for noise ROIs in addition to recovery curve limits is therefore a sensible method for obtaining comparable quantitative PET/CT data. Here, our COV measurements also form a better basis for comparing PET/CT systems with long and conventional axial FOVs in terms of image noise, than the NEMA sensitivity measurements [13, 14] that were originally not designed for long axial FOVs.

Two facts limit the informative value of this work: first, at the time of this study, the Biograph Vision Quadra was using a maximum ring distance of 85 (MRD 85), thus using maximally one fourth of the crystal rings for image acquisitions. While this gives the Quadra a homogenous sensitivity over the entire AFOV [14], a future update to the planned high sensitivity mode—using the full ring distance of 322 (MRD 322)—will bring forth an increase in sensitivity. Second, at the time of this study, the Biograph Vision Quadra was not equipped with continuous bed motion, and thus, this feature could not be studied here. However, the mentioned homogenous sensitivity over almost the full AFOV renders this currently a moot point. Beyond that, it can be safely assumed that the current results were mostly unaffected by the axial phantom position, and only at the very edge of the AFOV different results must be expected. However, once MRD 322 and continuous bed motion become available with the Biograph Vision Quadra, a re-evaluation of EARL compliance will become necessary.

Figure 7 also reveals a strong recovery with values above 0.5 for RC_max_ and RC_mean_ in the smallest sphere. It can therefore be assumed that the Biograph Vision Quadra is able to resolve even smaller structures. Its combination of high spatial resolution and high imaging sensitivity might require the introduction of smaller phantom spheres to analyze imaging properties at scales smaller than 10 mm, especially with the anticipated future sensitivity gains. To cover these smaller scales, we are planning to introduce smaller hot phantom spheres without cold walls [47], using additive manufacturing [48]. Employing long-lived phantoms will also allow evaluating the entire AFOV with a constant count rate in one single session.

## Conclusion

We conclude that EARL1 compliant reconstructions are possible with frames as short as 10 s duration on the Biograph Vision Quadra, provided that the reconstruction parameters are carefully chosen. An accordant reduction in patient dose might be also discussed instead of reducing frame duration. Optimal EARL1 compliant TrueX reconstructions for short frames were 6 iterations, 5 subsets, and 7.8 mm Gauss filtering. To achieve at least 30 s frames for EARL2 compliant measurements, it would be necessary to allow for more image noise.

While EARL 1 compliance proved very robust in terms of exposure, EARL2 compliance required a more careful selection of image reconstruction parameters. Especially, exposure was restricted to values above 1.2 MDecays/ml. Optimal EARL2 compliant TrueX reconstructions were achieved with 2 iterations, 5 subsets, and 3.6 mm Gauss FWHM post-reconstruction filtering in 5 min frames. Frames as short as 1 min were possible with these parameters. If, however, more matching EARL2 compliant RC values are intended between the Biograph Vision Quadra and for the Biograph Vision 600, we recommend using 4 iterations, 5 subsets, and 4.6 mm Gauss FWHM post-reconstruction filtering. This parameter combination will still yield exposure insensitive TrueX reconstructions.

From the three analyzed recovery metrics, RC_peak_ was the most stable with respect to exposure, but using the suggested reconstruction parameters, RC_max_ and RC_mean_ become also useful quantitative metrics under varying exposure.

Even though frames as short as 3 s do sometimes occur in dynamic studies, most clinical PET/CT acquisitions will most likely use longer frame durations. To this comes the caveat that the short time frames in a dynamic study are acquired immediately after tracer injection, at a moment where most of the activity is still located in the vascular system with little or no activity in the tissue of interest. Therefore, the quantification and measurability of a PET/CT image must be assessed non-stationarily, and simply relying on frame durations can lead to unquantifiable data. On the other side, a bolus or other regions with high uptake can become quantifiable even in short image frames. This is why we introduced the concept of exposure, where a VOI’s expected activity concentration determines the necessary acquisition duration.

It came somewhat to a surprise that EARL2 compliant exposure had an upper limit, meaning acquisition duration can actually become too long, and with it, the PET image becomes too noise-free. This certainly warrants a re-evaluation of EARL2 limits and possibly even the EARL procedures for total-body PET/CT systems: the lower achievable image noise justifies adjusting the bounds of the RC_max_ metric closer to accommodate high sensitivity PET/CT systems. EARL guidelines with a more flexible acquisition duration can also be discussed, mainly formulated in terms of exposure or acquired total counts. Harmonizing recovery values with respect to minimal or maximal attainable exposures would enhance comparability within frames of controlled dynamic studies and between dynamic studies across different PET/CT sites. This would make EARL, an already formidable harmonization strategy [28], even better.

## Data Availability

The datasets used and analyzed during the current study are available from the corresponding author on reasonable request.

## Abbreviations

AFOV: Axial field of view
COV: Coefficient of variation
CT: Computed tomography
EANM: European Association of Nuclear Medicine
EARL: EANM Research Ltd
FOV: Field of view
LSO: Lutetium oxyorthosilicate
NEMA: National Electrical Manufacturers Association
nRMSE: Normalized root-mean-square error
OSEM: Ordered subset expectation maximization
PET: Positron emission tomography
PSF: Point spread function
PVE: Partial volume effect
RC: Recovery coefficient
ROI: Region of interest
SNR: Signal-to-noise ratio
TOF: Time of flight
VOI: Volume of interest
